# Application of Injectable, Crosslinked, Fibrin-Containing Hyaluronic Acid Scaffolds for In Vivo Remodeling

**DOI:** 10.3390/jfb13030119

**Published:** 2022-08-13

**Authors:** Adél Hinsenkamp, Ágnes Fülöp, László Hricisák, Éva Pál, Kiara Kun, Aliz Majer, Viktória Varga, Zsombor Lacza, István Hornyák

**Affiliations:** 1 Institute of Translational Medicine, Semmelweis University, H-1094 Budapest, Hungary; 2 Orthosera Medical Zrt., H-1121 Budapest, Hungary; 3 Institute of Sport and Health Sciences, University of Physical Education, H-1121 Budapest, Hungary

**Keywords:** soft tissue implant, biocompatible, crosslinked hyaluronic acid, cryoprecipitate, injectable, in vivo remodeling, vascularization

## Abstract

The present research aimed to characterize soft tissue implants that were prepared with the use of crosslinked hyaluronic acid (HA) using two different crosslinkers and multiple reagent concentrations, alone or in combination with fibrin. The effect of the implants was evaluated in an in vivo mouse model, after 4 weeks in one group and after 12 weeks in the other. The explants were compared using analytical methods, evaluating microscopic images, and a histology analysis. The kinetics of the degradation and remodeling of explants were found to be greatly dependent on the concentration and type of crosslinker; generally, divinyl sulfone (DVS) resists degradation more effectively compared to butanediol diglycidyl ether (BDDE). The presence of fibrin enhances the formation of blood vessels, and the infiltration of cells and extracellular matrix. In summary, if the aim is to create a soft tissue implant with easier degradation of the HA content, then the use of 2–5% BDDE is found to be optimal. For a longer degradation time, 5% DVS is the more suitable crosslinker. The use of fibrin was found to support the biological process of remodeling, while keeping the advances of HA in void filling, enabling the parallel degradation and remodeling processes.

## 1. Introduction

There are three main factors in tissue engineering and regenerative medicine: cells, scaffolds, and chemical signals, such as cytokines and growth factors [1]. Stem cells are widely used in this field based on their ability to form de novo tissue and promote innate repair [2]. Scaffolds can serve to arrange them in three-dimensional architecture, providing structural support, and they act as an artificial extracellular matrix (ECM) [3]. Scaffolds can also be applied as carriers of chemical signals and promote their controlled release [4], while growth factors and cytokines contribute to the regulation of stem cell fate [5].

Scaffolds can be developed using synthetic or natural polymers. They can be biodegradable, non-biodegradable, and implanted with or without seeded cells; in this case, they can attract cells from the surrounding tissues after implantation. Synthetic scaffolds are advantageous because of their highly tunable properties, such as porosity, degradation time, and mechanical properties. In contrast, the advantages of natural scaffolds are their better interactions with cells, and they do not cause immune responses [1]. Many natural scaffolds are hydrogels, three-dimensional hydrophilic polymer networks, forming matrices, which can take up high volumes of water, up to a thousand times their dry weight [6]. Natural hydrogels can be fabricated using base materials such as hyaluronic acid [7], collagen [8], gelatin [9], silk-fibroin [10], chitosan [11], and alginate [12], among others, or as hybrid gels [13].

Hydrogels can be applied as soft tissue fillers in the case of significant tissue loss, congenital defects, disease, trauma, or aging [1]. Injectability is an essential property in hydrogels, as injectable gels can precisely fill up tissue cavities; they are applicable in practically any organ and tissue. Moreover, in this case, the implantation process is minimally invasive [14].

Hyaluronic acid (HA) is an optimal base material for soft tissue engineering applications based on its physical and biological properties. HA is an unbranched, non-sulphated glycosaminoglycan consisting of repeating units of N-acetyl D-glucosamine and D-glucuronic acid, capable of reaching a very high molecular weight (10^8^ Da) [15]. It appears in different molecular weights in the mammalian body, which affects its biological properties: low molecular weight HA (under 100 kDa) induces inflammatory chemokines, immune responses, and angiogenesis, while high molecular weight HA is reported to possess anti-inflammatory, immunosuppressive, and anti-angiogenic properties [16,17]. It can naturally be found in the skin, synovial fluid, vitreous body, umbilical cord, joints, tendons, sheaths, pleura, and the pericardium; a human body of 70 kg contains 15 g of hyaluronic acid [18]. Commercial HA can originate from animal sources, such as rooster comb; however, HA used in medical applications is most commonly produced by biotechnology and microbial fermentation. Ideal HA-producing microorganisms do not secrete toxins and can make at least 10^6^ Da HA. Microorganism-derived HA is biocompatible with the mammalian body since the structure of HA is highly conserved between different species [15].

HA degrades in vivo rapidly by hyaluronidase enzymes and oxidative damage [15]. In the case of soft tissue fillers, when rapid degradation is a disadvantage, HA can be physically or chemically modified to prolong its presence after implantation. The concentration of the crosslinker reagents affects the degradation time, water uptake capacity, and the stiffness of the hydrogels [19]. The in vitro degradation was analyzed in our previous work using both divinyl sulfone (DVS) and butanediol diglycidyl ether (BDDE) in two relevant concentrations. We found that BDDE is slightly more resistant to hyaluronidase-initiated degradation compared to DVS if they are used in the same concentration [20]; however, further modification can enable significantly more resistance, as described. In the case of biodegradable hydrogels, the aim is to create a scaffold which resists fast degradation; however, it can be remodeled by attaching cells while new tissue is formed and, preferably, it promotes the vascularization of the implanted hydrogel [20].

Native and crosslinked long-chain HA is highly biocompatible; however, it was found to be rather bioinert when applied alone [21]. It does not induce cellular attachment and proliferation on the hydrogel [22]. Hybrid hydrogels may be fabricated to promote cellular attachment, for example, with fibrin [20,23]. Fibrinogen and fibrin are essential for several biological functions in the human body, for example, in hemostasis and wound healing [24,25]. Fibrin-based scaffolds are also widely used in regenerative medicine, for example, in dentistry [26], based on their excellent ability to facilitate cell attachment, besides their growth factor content [27,28].

The fibrinogen level can be enriched in human plasma by cryoprecipitate isolation [29]. In this case, its concentration can be multiplied, together with other proteins, which have limited solubility at a low temperature: factor VIII, von Willebrand factor, factor XIII, and fibronectin [30,31]. Growth factors are also present in cryoprecipitate; however, their concentration is not elevated compared to fresh frozen plasma (FFP) [28].

In our previous investigations, we discovered that the natural clotting process that occurs in whole blood also occurs in plasma and cryoprecipitate, as well as in purified fibrinogen, and these can be combined with biomaterials suitable for scaffold preparation [20,28]. In the present study, we aimed to test the biocompatibility, biodegradability, remodeling, and vascularization of differently crosslinked fibrin containing hyaluronic acid hydrogels in vivo in C57BL/6 mice, based on a previous study [20]. The cell attachment capacity of the hydrogels was improved by fibrin polymerization into the crosslinked HA matrices. The fibrinogen originated from human FFP, and its concentration in the plasma was augmented by cryoprecipitate isolation [20]. Butanediol diglycidyl ether and divinyl sulfone were used as crosslinking reagents, two chemical crosslinkers acting on the hydroxyl groups of HA [32,33]. They are already well-known and used in existing products [34,35], which enables our hydrogels to be regulated as a medical device in the future, as we aim to develop a commercialized product after further investigation [36,37].

## 2. Materials and Methods

### 2.1. Hydrogel Preparation

The hydrogels were prepared as described in our previous work [20], briefly: 1.34 MDa molecular weight sodium hyaluronate (Contipro, Dolní Dobrouč, Czech Republic) was crosslinked using DVS (abcr, Karlsruhe, Germany) or BDDE (Sigma-Aldrich, St. Louis, MO, USA). The crosslinkers were used in 2 or 5 V/V% concentrations (20 or 50 µL), added to 1 mL 1 w% NaOH solution and poured onto 133 mg of freeze-dried sodium hyaluronate. The mixture was immediately vortexed and centrifuged at 1700× *g* for 2 min to obtain a flat hydrogel. The gels were allowed to crosslink for 24 h, then washed with 80 mL of distilled water three times every 24 h. The medical devices that contain crosslinked HA are generally sterilized using heat sterilization, thus, the samples were autoclaved at 121 °C for 20 min. The sterilized gels were frozen at −80 °C and freeze-dried at −55 °C and 5 Pa.

### 2.2. Cryoprecipitate Isolation

Human fresh frozen plasma, isolated by plasmapheresis, was purchased from Hungarian National Blood Transfusion Service, Budapest, Hungary (OVSz). The plasma was kept at −80 °C for at least 24 h and slowly thawed at 3 °C. It was centrifuged at 3260× *g* for 12 min at 3 °C. Then, 60 V/V% of the supernatant was removed, and the precipitate was dissolved in the remaining 40 V/V% of the plasma. The plasma product contained 4.68 g/L of fibrinogen [28].

### 2.3. Fibrin Addition

Each freeze-dried hydrogel was supplemented with 4 mL of cryoprecipitate, which was activated by adding 800 µL 1 w% sterile Calcium D-gluconate (anhydrous, Sigma-Aldrich, St. Louis, MO, USA) solution. The cryoprecipitate was activated and immediately poured onto the HA matrices. The freeze-dried hyaluronic acid absorbs the activated cryoprecipitate, and clotting can take place inside the spongious hydrogel, resulting in entrapped fibrin fibers inside the HA scaffolds.

### 2.4. Homogenization

Fibrin crosslinking was allowed to take place for 24 h at 4 °C, then the fibrin-containing hydrogels were homogenized for 5 min using a homogenizer (Tissueruptor; Qiagen, Hilden, Germany). Homogenization was facilitated by adding 4 mL of plasma supernatant to each fibrin-containing hydrogel. As a control, the crosslinked freeze-dried hydrogels were homogenized without fibrin; in this case, homogenization was facilitated by adding 4 mL of sterile distilled water to each hydrogel. When 2% of BDDE-containing hydrogel was homogenized, 4 mL of distilled water was insufficient to produce an injectable homogenizate. According to our previous study, this type of hydrogel has the highest swelling ratio [20], thus, it needed 6 mL of distilled water for homogenization instead of 4 mL. After homogenization, the aseptically produced hydrogels were filled into sterile syringes.

### 2.5. HA Injection to Mice

**Figure 1:**
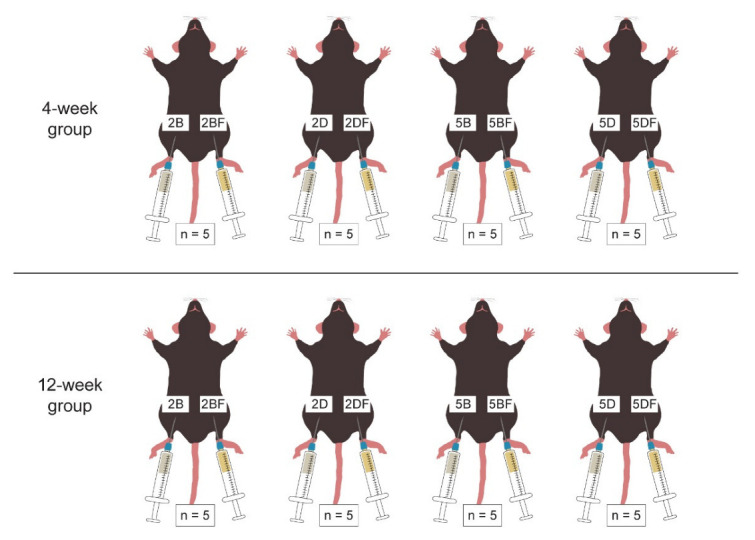
Distribution of scaffold types and implantation time groups in the in vivo model.

Two-month-old male C57BL/6 mice were involved in our experiments, according to the guidelines of the Hungarian Law of Animal Protection (XXVIII/1998). All procedures were approved by the National Scientific Ethical Committee on Animal Experimentation (PEI/001/2706-13/2014, approval date: 17 December 2014, and PE/EA/00487-6/2021, approval date: 30 December 2021). The animals were housed at a constant temperature with a 12-h light/12-h dark cycle and had ad libitum access to food and water. At the beginning of the experiment, they were anesthetized with 2% isoflurane, and 200 µL of homogenized hydrogel was injected subcutaneously to the abdominal site of the hindlimb. The water containing control gel was injected into the right limb, and the fibrin containing gel was injected into the left limb. Figure 1 demonstrates the scaffolds: types 1 and 2 were 2% BDDE (2B, and 2BF)-containing hydrogels; types 3 and 4 received 5% BDDE (5B, and 5BF) crosslinked gel; types 5 and 6 were 2% DVS (2D, and 2DF)-containing gels; and types 7 and 8 were HA-crosslinked with 5% DVS (5D, and 5DF). Each scaffold type was injected into five–five mice: five were examined after four weeks (4-week group), and the remaining five mice were observed twelve weeks after the implantation (12-week group).

### 2.6. Harvesting the Scaffolds

After four weeks, the 20 animals in the 4-week group were euthanized by cervical dislocation, and their hind limbs were depilated. An incision was made to expose the hydrogels, which were observed by a light microscope (Leica M80; Leica Microsystems, Wetzlar, Germany) and the implants were removed. The procedure was repeated after twelve weeks with the 20 animals in the 12-week group. After the scaffolds were removed, they were fixed in 4% formaldehyde, their weights were measured, and they were sent for histology measurements.

### 2.7. Hematoxylin-Eosin (H-E) Staining

The harvested hydrogels were dehydrated and cleared in water, ethanol, and xylol. Then, the samples were embedded in paraffin and cut into 2.5 µm thick sections. They were dyed using Mayer’s hematoxylin and 1% eosin.

The images of H-E staining were evaluated using ImageJ software, area percentage and vascularization were quantified in grayscale and RGB stack, respectively, and the images were normalized to be comparable. The H-E staining was quantified using color threshold differences that were found to be characteristic for the area of HA and the area of cells + ECM.

### 2.8. Statistical Analysis

The statistical analysis was performed with a *t*-test, one-way analysis of variance (ANOVA) with Tukey’s post hoc test, or two-way ANOVA with Bonferroni post-tests to compare the means of samples using Prism 7 software. The significance level was *p* < 0.05, where * means that *p* is between 0.01 and 0.05, ** means that p is between 0.01 and 0.001, and *** means that *p* is lower than 0.001, and the data are presented as mean ± standard error of the mean.

## 3. Results

### 3.1. Weight Measurement

**Figure 2:**
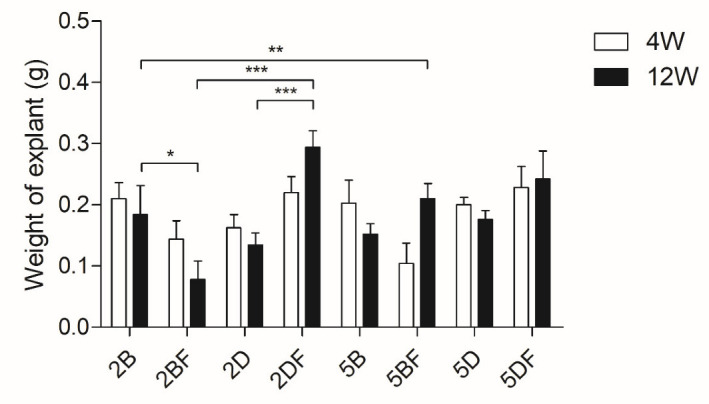
Weight measurement of the explants 4 and 12 weeks after implantation. 2B: 2% BDDE-crosslinked HA; 2BF: 2% BDDE-crosslinked HA with fibrin; 2D: 2% DVS-crosslinked HA; 2DF: 2% DVS-crosslinked HA with fibrin; 5B: 5% BDDE-crosslinked HA; 5BF: 5% BDDE-crosslinked HA with fibrin; 5D: 5% DVS-crosslinked HA; 5DF: 5% DVS-crosslinked HA with fibrin. The significance level was *p* < 0.05, where * means that *p* is between 0.01 and 0.05, ** means that *p* is between 0.01 and 0.001, and *** means that *p* is lower than 0.001, and data are presented as mean ± standard error of the mean. There were no significant differences between the 4-weeks and 12-weeks groups; however, significance levels were indicated in the figure between groups where only one parameter was different. All significance levels between different groups are shown in Table 1.

After the injected implants were removed, the weights of the explants were measured on an analytical balance after the four- and twelve-week experiments to obtain information about the degradation and remodeling of the scaffolds. The hypothesis was that the 2% crosslinker-containing scaffolds would be more degraded than the 5% crosslinker-containing ones. After 12 weeks of implantation, the matrices were expected to be more degraded and more remodeled than after 4 weeks. We also expected faster degradation but more advanced remodeling in the case of the fibrin-containing scaffolds compared to the control gels. The comparison was made by measuring the weight of the explants (Figure 2), and the data, which were available after the histology analysis.

There was no significant difference between the weight of the explants, whether those removed after 4 weeks or 12 weeks if they contained the same type of scaffold. The lowest weight belonged to the 2% BDDE and fibrin-containing scaffold type (2BF), and there were significant differences in the weight of the explants between the scaffold types and groups, as visible in Table 1.

In most cases, the DVS-containing scaffolds weighed more than the corresponding BDDE-containing scaffolds, and the highest weight was in the case of DVS and fibrin-containing scaffolds (Figure 2).

### 3.2. Relative Cross-Section Area of the Implants

**Figure 3:**
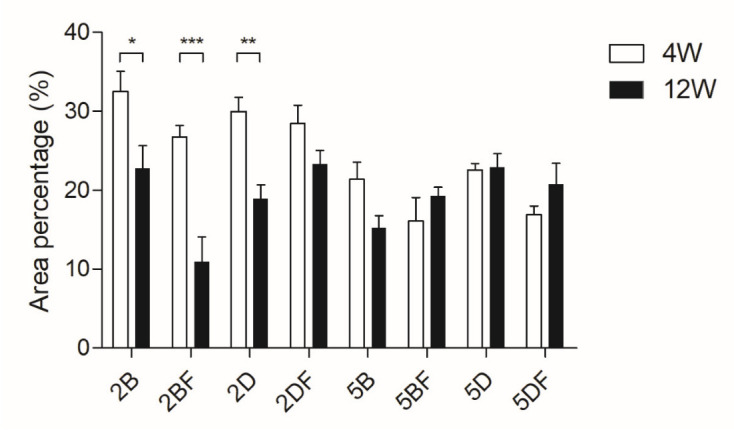
Relative cross-section area of the implants that were removed after either 4 weeks or 12 weeks and underwent histology analysis. The significance level was *p* < 0.05, where * means that *p* is between 0.01 and 0.05, ** means that *p* is between 0.01 and 0.001, and *** means that *p* is lower than 0.001, and data are presented as mean ± standard error of the mean.

The samples also underwent a histology analysis; the area percentage was compared within and between the groups using the area fraction calculation function of ImageJ in grayscale 8-bit mode. The results can be observed in Figure 3.

We found a significant difference between 4 weeks and 12 weeks of implantation in the case of 2% BDDE-crosslinked (both control and fibrin-containing) scaffold types and 2% DVS-crosslinked (control) scaffold types.

The significant differences between the scaffold types and groups are collected in Table 2.

Generally, the cross-section area was larger in the 2% crosslinker-containing scaffolds, which is in accordance with the higher swelling ratio due to the lower crosslinker content. However, this trend seems to have changed after 12 weeks, especially in the case of 2BF, where similarly to the weight diagram, high degradation is visible.

It needs to be pointed out that in the case of 5% crosslinker-containing scaffold types, no significant cross-section decrease was visible between the 4-week and 12-week groups. In the case of 5DF, an increase can even be observed after 12 weeks, compared to 4 weeks of implantation, which probably means that degradation happens parallelly with remodeling. However, the size and shape of the implant remained intact.

### 3.3. Microscopic Images of the Implants

**Figure 4:**
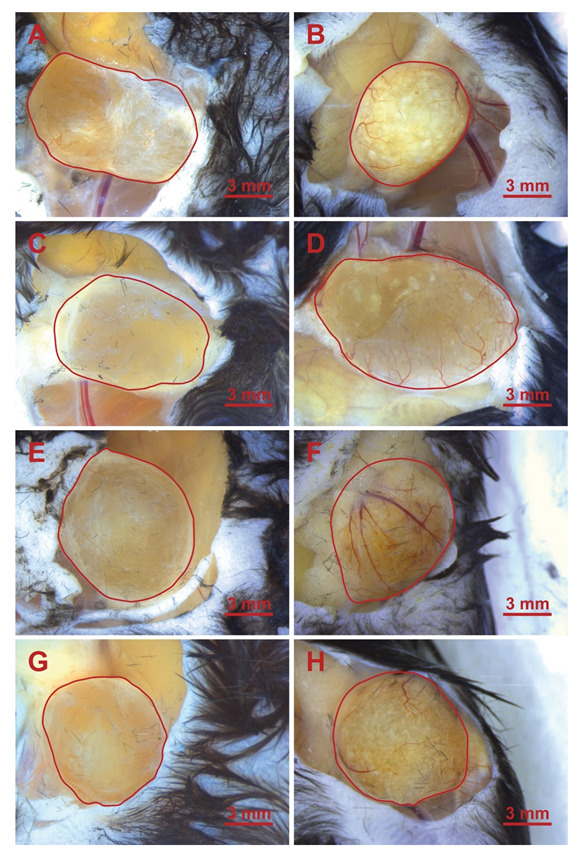
HA implants in the leg of the mice after 4 weeks. (**A**): 2B; (**B**): 2BF; (**C**): 2D; (**D**): 2DF; (**E**): 5B; (**F**): 5BF; (**G**): 5D; (**H**): 5DF.

**Figure 5:**
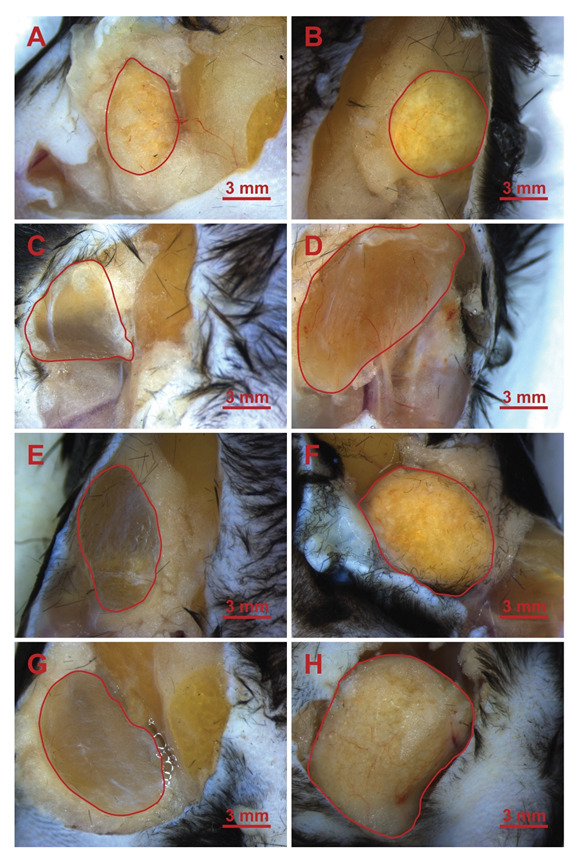
HA implants in the leg of the mice after 12 weeks. (**A**): 2B; (**B**): 2BF; (**C**): 2D; (**D**): 2DF; (**E**): 5B; (**F**): 5BF; (**G**): 5D; (**H**): 5DF.

The implants were observed under a light microscope, and representative images were taken of the implants after the animals were sacrificed. The implant areas are highlighted in red; the 4-week long implantation images are visible in Figure 4, and the 12-week long ones are visible in Figure 5. In the left-side images, the native (control) hyaluronic acid-based scaffolds are visible, which were crosslinked, but did not contain fibrin coating. In the right side images, the fibrin-containing scaffold is visible.

### 3.4. Evaluation of Vascularization

**Figure 6:**
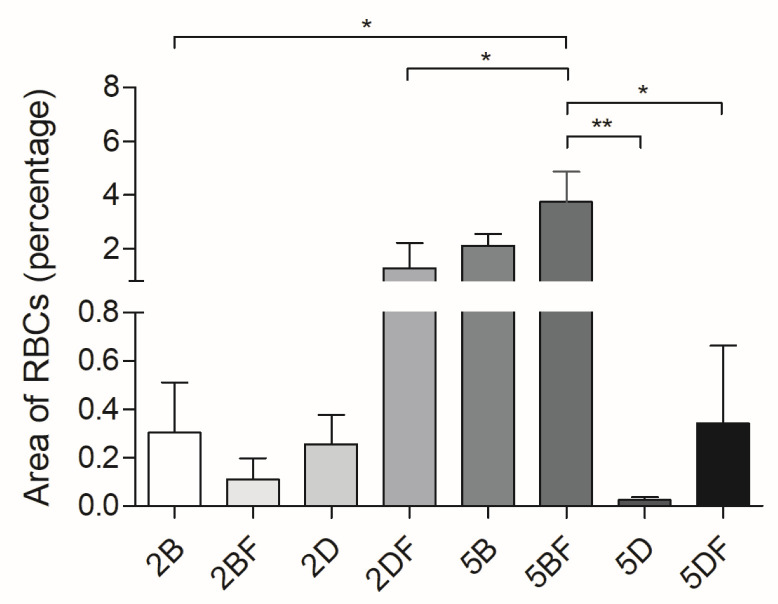
Red blood cell area percentage of the cross-sections of the stained explants 12 weeks after implantation. The significance level was *p* < 0.05, where * means that *p* is between 0.01 and 0.05, and ** means that *p* is between 0.01 and 0.001. Data are presented as mean ± standard error of the mean.

The explants were visualized under a light microscope, and the distribution of formed blood vessels was calculated using ImageJ. In the case of 4 weeks of implantation, the difference in blood vessel formation was not significant; however, there were significant differences after 12 weeks of implantation. The results are presented in Figure 6.

### 3.5. Histological Analysis

**Figure 7:**
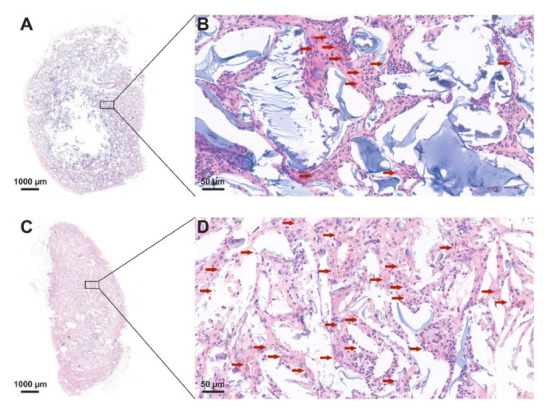
Representative red blood cell distribution of the cross-sections of the stained explants: (**A**) and (**B**): 5DF, and (**C**) and (**D**): 5BF 12 weeks after implantation.

The implants were embedded in paraffin, and the cross-sections were stained using hematoxylin-eosin. A representative image is shown in Figure 7, where the presence of red blood cells is indicated with red arrows, and the 5BF and 5DF types of scaffolds are compared.

The representative image shows that in accordance with Figure 6, vascularization was enhanced in the 5BF scaffold type compared to the 5DF scaffold type.

### 3.6. The Relative Area of HA and Cells + Extracellular Matrix

**Figure 8:**
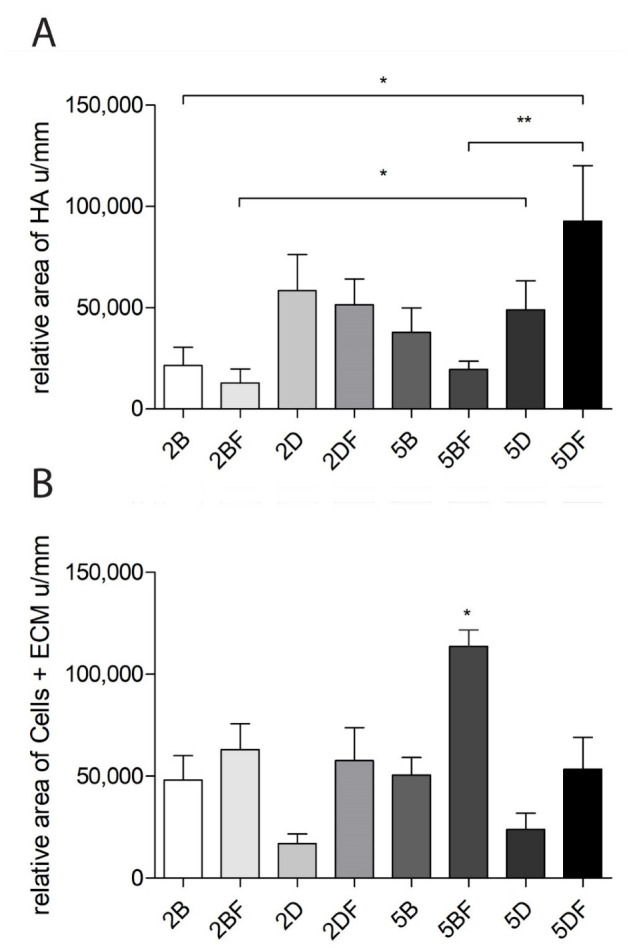
(**A**) The remaining HA area in the stained cross-sections after 12 weeks. (**B**) Area of the formed cells and ECM in the stained cross-sections after 12 weeks. The significance level was *p* < 0.05, where * means that *p* is between 0.01 and 0.05, and ** means that *p* is between 0.01 and 0.001. Data are presented as mean ± standard error of the mean.

The relative area of HA and cell + extracellular matrix was also compared between the scaffold types. At the same time, no significant differences were visible after 4 weeks of implantation (data not shown). After 12 weeks, there were significant differences between the areas of the remaining HA (Figure 8A) and the area infiltrated by cells and the extracellular matrix (Figure 8B).

The remaining HA area was the highest when 5% DVS crosslinker was used, and the scaffold contained fibrin (5DF). Thus, it can be stated that the DVS-containing scaffolds are generally more resistant to degradation than BDDE-containing hydrogels with the same crosslinker concentration. Moreover, 2% DVS scaffolds were more resistant to degradation than those containing 5% BDDE.

Interestingly, the area covered by cells and ECM was the highest in the scaffolds that contained 5% BDDE crosslinker and fibrin (5BF). The implants were significantly more infiltrated by cells and ECM in the case 5BF. The tendency was that fibrin-containing scaffolds contained more cells and ECM, compared to the scaffolds crosslinked with the same crosslinker, but which did not contain fibrin.

## 4. Discussion

Hyaluronic acid was crosslinked using BDDE or DVS in 2 or 5 V/V% concentrations, and the hydrogels were supplemented with human plasma to promote cellular attachment. These scaffolds were homogenized and injected subcutaneously into the hindlimb of mice. As a control, crosslinked HA was implanted without fibrin. The scaffolds were harvested and examined after 4 and 12 weeks. The explants were observed under a light microscope, their weight was measured, and they underwent hematoxylin-eosin staining and a histological analysis.

The crosslinking clearly enabled a higher resistance to degradation: a similar investigation used hybrid HA-ALG (alginate) scaffolds which degraded over 8 weeks of implantation without covalent crosslinking [38]. In our case, there was no significant difference in the weights of the implants within the same type of scaffolds after 4 or 12 weeks of implantation. Furthermore, there were only a few significant differences in the weights of the explants that were removed after 4 weeks of implantation between either of the eight scaffold types. However, there were significant differences between both 2 and 5% DVS crosslinker containing HA scaffolds after 12 weeks in the case when fibrin was supplemented, compared to the native implant (2D vs. 2DF ***; 5D vs. 5DF *). Thus, the presence of fibrin caused significant difference in both cases: the fibrin-containing scaffolds had a larger weight. 

Interestingly, the mean weight in the eight scaffold types after 4 weeks was 183.9 mg; after 12 weeks, it was 183.8 mg, which is almost identical. In a similar study, 20.02 µL of DVS was added to 68 mg of HA and dissolved in 1.68 mL solution. That mixture lost more than half of the original volume after 12 weeks of implantation; however, the DVS concentration in their solution may have been lower (1.2 V/V%), compared to our setup [39]. Based on Figure 8A,B, the remaining HA, and the formed cellular components (cells + ECM) do show significant differences after 12 weeks of implantation between the scaffold types; this can probably be explained by the dynamics of degradation and remodeling that seem to be happening parallelly in our case.

Figure 4 and Figure 5 representative images show the different scaffold types implanted in the phase when the animals were sacrificed, and the implants were ready to be removed. Generally, it is visible that the control implants, which did not contain fibrin (they can be seen on the left side in every row) are more transparent, which is similar to the results of Yang, et al., who used 0.4% BDDE [40] and discovered low cell and ECM content within the implants. Those containing fibrin (in the right-side images) are hazier and grainier, indicating vascularization and the presence of more solid phased materials, supporting the results that can be seen in Figure 7 and Figure 8B for the 12-week group.

The porosity can also be an interesting factor; using 2% BDDE in the scaffolds probably leads to weaker intermolecular bonds and, thus, a larger swelling ratio due to the larger gaps between the HA chains. Interestingly, this difference is not that obvious based on the weight differences (Figure 2), which may partly be since ECM can affect swelling compared to water [41]. The histology analysis allowed us to visualize that 2% crosslinker-containing scaffolds have a larger diameter, compared to those containing 5% crosslinker, especially after 4 weeks of implantation (Figure 3), based on the H-E staining evaluation. However, this difference becomes smaller after 12 weeks of implantation.

Based on the results of Figure 8A, we found that generally, the degradation of BDDE-crosslinked scaffolds was faster in all the 12-week groups compared to the degradation of DVS-containing hydrogels when the crosslinker was used in the same concentration. According to the evaluation with the use of ImageJ, it is visible that both 2% and 5% DVS crosslinker enables greater resistance to degradation compared to BDDE used in the same concentrations, which can probably be explained by the finding that a more complicated crosslinker results in slower degradation [42]. In our case, DVS is capable of reacting with water, HA itself, and in combination with these; thus, it probably creates heterogenic crosslinking, which can slow degradation.

The presence of fibrin seems to be important in the case when we evaluated the area covered by cells in the scaffolds, as can be seen in Figure 8B. In all the samples of the 12-week group, the fibrin-containing scaffold had a larger cell and ECM content in the cross-section of the implants based on the evaluation of the H-E staining.

It can also be observed that in almost all samples of the 12-week group, the fibrin-containing implant developed more blood vessels than the control ones. It means that the fibrin content probably enhanced the number of cells and ECM and the vascularization of the implants, a theory which is supported by the scientific literature [25,43]. Surprisingly, this did not lead to significant differences after 4 weeks, only after 12 weeks. The difference can easily be observed visually in Figure 7, where fibrin-coated, 5% crosslinker-containing scaffolds are shown after 12 weeks. The upper one is crosslinked with DVS (5DF), and the lower one contains BDDE (5BF). The arrows indicating the formation of blood vessels are in good accordance with Figure 6 and Figure 8B, indicating that 5BF is the best candidate for remodeling. The upper one, 5DF, contains fewer blood vessels and is generally more “blueish”, which is in good accordance with Figure 2 and Figure 8A, indicating that 5DF is the best candidate for durable implantation, with the slowest HA degradation.

## 5. Conclusions

We aimed to investigate the short (4 week) and long (12 week) effect of crosslinked hyaluronic acid that was crosslinked with two different crosslinkers (BDDE and DVS) in two different concentrations (2 and 5%). These scaffolds were supplemented with fibrin from FFP to enhance cell attachment and vascularization. The two best candidates were found to be 5% DVS and 5% BDDE crosslinker-containing HA scaffolds that were supplemented with fibrin. Both candidates enhanced the amount of ECM and cells that were present in the scaffolds and led to better vascularization and remodeling compared to the same HA scaffold types that did not contain fibrin. However, we came to the conclusion that these two types of scaffolds can probably serve different purposes as soft tissue implants. Thus, if the intended use is faster degradation, then the use of 2–5% BDDE is advised, and for a longer degradation (probably well over 90 days), 5% DVS is the suggested crosslinker. However, the use of fibrin is always a good choice to enhance remodeling.

## Figures and Tables

**Table 1:** Significant weight differences between the scaffold types that were removed after 4 weeks or 12 weeks. The significance level was *p* < 0.05, where * means that *p* is between 0.01 and 0.05, ** means that *p* is between 0.01 and 0.001, and *** means that *p* is lower than 0.001, and data are presented as mean ± standard error of the mean.

Scaffold Type	Scaffold Type	Time Group (Week)	Weight Difference	Scaffold Type	Scaffold Type	Time Group (Week)	Weight Difference
2B	2BF	12	*	2D	2DF	12	***
2B	2DF	12	*	2D	5DF	12	*
2B	5BF	4	*	2DF	5B	12	**
2BF	2DF	12	***	2DF	5BF	4	*
2BF	5BF	12	**	2DF	5D	12	*
2BF	5D	12	*	5BF	5D	4	*
2BF	5DF	12	***	5BF	5DF	4	**

**Table 2:** Significant cross-section area differences between the scaffold types that were removed after 4 weeks or 12 weeks. The significance level was *p* < 0.05, where * means that *p* is between 0.01 and 0.05, ** means that *p* is between 0.01 and 0.001, and *** means that *p* is lower than 0.001, and data are presented as mean ± standard error of the mean.

Scaffold Type	Scaffold Type	Time Group (Week)	Area Difference	Scaffold Type	Scaffold Type	Time Group (Week)	Area Difference
2B	2BF	12	***	2BF	5DF	4	**
2B	5B	4	*			12	**
		12	***	2D	5B	4	*
2B	5BF	4	***	2D	5BF	4	***
2B	5D	4	**	2D	5D	4	*
2B	5DF	4	***	2D	5DF	4	***
2BF	2D	12	*	2DF	5B	4	*
2BF	2DF	12	***			12	*
2BF	5BF	4	**	2DF	5BF	4	***
		12	*	2DF	5D	4	***
2BF	5D	12	***	5B	5D	12	*

## Data Availability

The data presented in this study are available on request from the corresponding author.
